# Portable Perimetry Using Eye-Tracking on a Tablet Computer—A Feasibility Assessment

**DOI:** 10.1167/tvst.8.1.17

**Published:** 2019-02-05

**Authors:** Pete R. Jones, Nicholas D. Smith, Wei Bi, David P. Crabb

**Affiliations:** 1 Division of Optometry and Visual Science, School of Health Sciences, City, University of London, London, UK

**Keywords:** glaucoma, visual fields, static perimetry, eye-movements

## Abstract

**Purpose:**

Visual field (VF) examination by standard automated perimetry (SAP) is an important method of clinical assessment. However, the complexity of the test, and its use of bulky, expensive equipment makes it impractical for case-finding. We propose and evaluate a new approach to paracentral VF assessment that combines an inexpensive eye-tracker with a portable tablet computer (“Eyecatcher”).

**Methods:**

Twenty-four eyes from 12 glaucoma patients, and 12 eyes from six age-similar controls were examined. Participants were tested monocularly (once per eye), with both the novel Eyecatcher test and traditional SAP (HFA SITA standard 24-2). For Eyecatcher, the participant's task was to simply to look at a sequence of fixed-luminance dots, presented relative to the current point of fixation. Start and end fixations were used to determine locations where stimuli were seen/unseen, and to build a continuous map of sensitivity loss across a VF of approximately 20°.

**Results:**

Eyecatcher was able to clearly separate patients from controls, and the results were consistent with those from traditional SAP. In particular, mean Eyecatcher scores were strongly correlated with mean deviation scores (*r^2^* = 0.64, *P* < 0.001), and there was good concordance between corresponding VF locations (∼84%). Participants reported that Eyecatcher was more enjoyable, easier to perform, and less tiring than SAP (all *P* < 0.001).

**Conclusions:**

Portable perimetry using an inexpensive eye-tracker and a tablet computer is feasible, although possible means of improvement are suggested.

**Translational Relevance:**

Such a test could have significant utility as a case finding device.

## Introduction

Measuring visual field loss is a key component of an eye examination. It is a particular cornerstone of glaucoma assessments, where optic nerve damage typically results in asymptomatic visual field loss that can only be detected by perimetric examination.^1^ When deployed appropriately, standard automated perimetry (SAP) can be used successfully to manage visual field loss and evaluate treatment efficacy.^2^ However, current SAP devices lack portability and are expensive. This makes them impractical in some settings, and restricts in particular their use in glaucoma case finding. Furthermore, many people find visual field examination by SAP challenging or uncomfortable. Indeed, even experienced test takers describe feelings of anxiety when faced with a visual field examination.^3^ Sometimes it is best to describe a problem in lay terms: pressing a button to detect spots of light in a large white bowl whilst trying to keep your eye and head perfectly still is simply an unnatural task.

A portable, inexpensive, and user-friendly device would allow for more effective visual field screening, particularly in resource-poor or hard to reach communities. This in turn could help to improve detection rates for diseases such as glaucoma (which are currently poor^1^,^4^), and may lead to fewer patients presenting to hospital eye service with already advanced visual field loss.^5^–^7^

One way to make perimetry easier and more comfortable is to use eye- and head-tracking technology. If the position of eye and head can be monitored accurately, then stimuli can be presented relative to the current point of fixation, irrespective of where the patient is looking. Furthermore, stimuli can be scaled to be a constant size on the retina, irrespective of viewing distance. This removes completely the need for chin rests or fixation crosses. In addition, the use of eye-tracking further obviates the need for any button-pressing or detailed task instructions, since eye-movements toward transient stimuli are largely reflexive, and occur robustly even in infants^8^–^10^ and individuals with cognitive impairments.^11^ Accordingly, a number of groups have recently developed eye-tracking based perimetry,^12^–^17^ capable, for example, of discriminating between healthy and glaucomatous eyes.^14^ However, to date these systems still suffer from many of the same limitations regarding portability and cost.

One way to make perimetry more portable and inexpensive is to exploit modern tablet computers.^18^,^19^ Thus, a number of tablet-based apps have recently been developed for measuring visual field loss,^20^–^26^ as well as other aspects of visual function such as visual acuity (VA),^27^–^30^ contrast sensitivity (CS),^31^–^35^ foveal defects,^36^ and stereopsis.^37^ The hope is that these tests could be used as an alternative to traditional, more expensive tests, or could allow patients to be monitored more regularly from the comfort of their own homes. This could bring considerable benefits in terms of reduced healthcare costs, an improved patient experience, and the ability to detect high-risk individuals more quickly than traditional (i.e., annual or biannual) monitoring regimens.^20^ However, the lack of eye- and head-tracking is an acknowledged limitation of current tablet perimeters.^22^ To ensure that stimuli are accurately localized on the retina, the patient is required to keep their head steady and their fixation constant: tasks that many patients find difficult and unnatural.^38^ And, unlike SAP, a technician is not on hand to ensure compliance. These factors are likely to severely limit the accuracy and reliability of any test results.

The current study represents a first attempt to bring together the best of these two strands of research, by combining an ordinary tablet computer with an inexpensive “clip-on” eye-tracker. The resultant test, which we call “Eyecatcher,” is not intended as a like-for-like replacement for SAP, but is instead proposed as a portable, inexpensive, rapid, and easy-to-use case finding device. Unlike SAP, eye-movements are permitted, and no button-pressing or head restraint is required: the participant is required only to look at the tablet screen and follow a dot with their eye. Also unlike standard SAP, stimuli were of fixed luminance (suprathreshold perimetry), as the test was only intended to detect clinically meaningful defects.

In this report, we present Eyecatcher and compare it with SAP in people with glaucoma and visually healthy peers. Specifically, we examined whether the two tests gave concordant results, and evaluated their relative speed, completion rates, and levels of user-satisfaction. We also identify limitations of the approach and this current implementation.

## Methods

### Participants

Participants were 12 patients with diagnosed glaucoma (median age, 70 years), and six age-similar controls (median age, 76 years; see the Table). Patients were recruited from clinics at Moorfields Eye Hospital NHS Foundation Trust, London. All patients had an established (4+ years) clinical diagnosis of chronic open-angle glaucoma in both eyes, and were being treated. A deliberate attempt was made to recruit a sample of patients with a wide range of disease severity according to visual field loss (see the Table). Patients were purposely not recruited if they had any ocular disease other than glaucoma (except for an uncomplicated lens replacement cataract surgery). Age-similar controls were recruited from the City, University of London Optometry Clinic; this is a primary care center where people routinely receive a full eye examination, which includes measurement of VA, refraction, binocular vision assessment, pupil reactions, slit-lamp assessment of the anterior eye, measurement of intraocular pressure, visual field assessment and indirect ophthalmoscopy of the macula, optic nerve head, and peripheral retina. All participants had taken part in studies in our research laboratory before. Institutional Ethics Committee approval was obtained, and the research adhered to the tenets of the Declaration of Helsinki. Written informed consent was obtained prior to all examinations.

**Table:** Participant ages and clinical screening measures. VA was assessed using an ETDRS chart, and is reported in logMAR (BL, Bare Light perception only). Visual fields were assessed using SAP (24-2 SITA standard) and is reported in MD (dB)—see also Figure 3 for pointwise data. CS was measured using Pelli Robson charts, and is reported in logCS.

### Vision Testing

Standard visual fields were measured in both eyes using the Humphrey Field Analyzer (HFA; Carl Zeiss Meditec, CA) with the central standard 24-2 Swedish Interactive Testing Algorithm. The Glaucoma Hemifield Test (GHT) was “outside normal limits” for all eyes in all patients and “within normal limits” for all eyes in all controls.^39^ If any of the visual fields were flagged by the HFA output as “unreliable” (as assessed by false positives, false negatives, or poor fixation measures), then the test was repeated. HFA mean deviation (MD) values were used as a measure of overall glaucomatous disease severity in the patient group.

Corrected monocular VA was measured using an Early Treatment Diabetic Retinopathy Study (ETDRS) chart. CS was measured in log units with a Pelli-Robson chart. The Oculus C-Quant straylight meter (Oculus GmbH, Wetzlar, Germany) was used to measure abnormal light scattering in the eye media, in order to eliminate significant media opacity and other lens type artefacts as confounding ocular conditions; all participants were required to be within “normal limits” for this test.

### The Novel Test (Eyecatcher)

#### Apparatus

**Figure 1:**
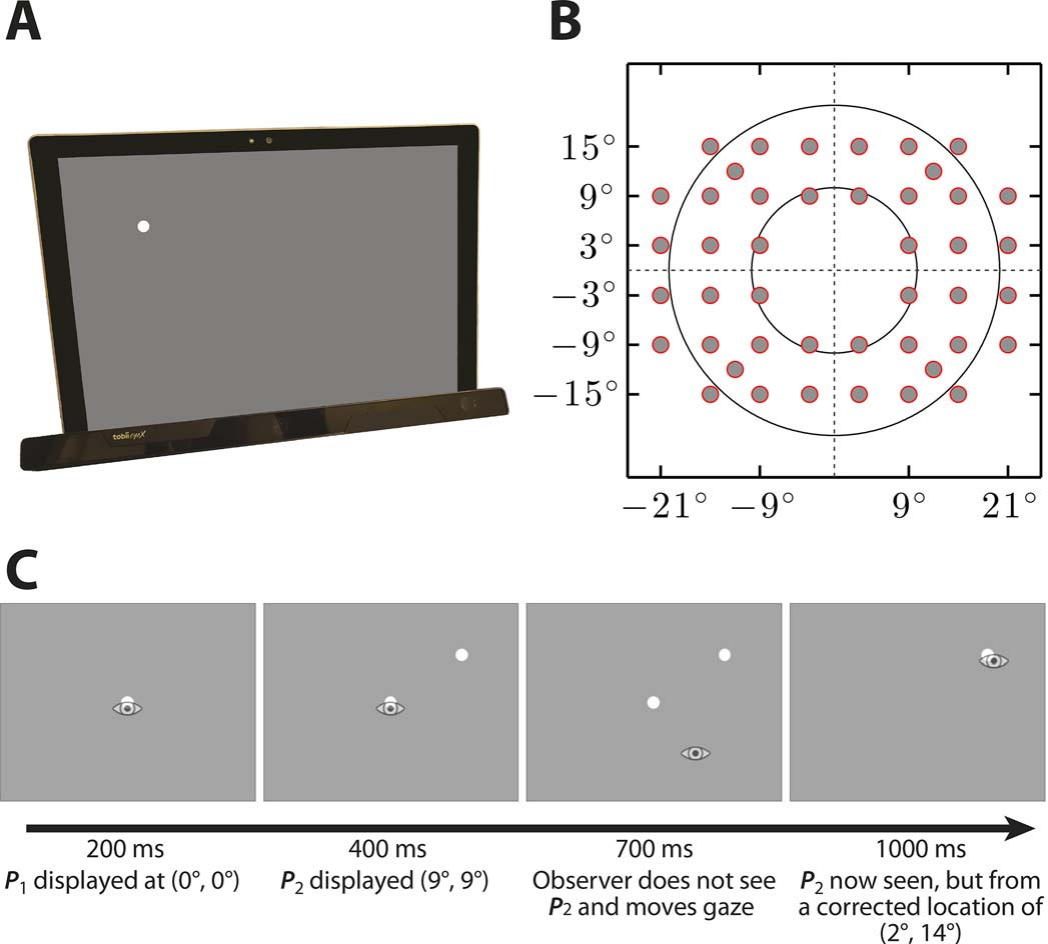
Eyecatcher Methods. (A) The key test equipment, comprised of a Microsoft Surface Pro 3 tablet (viewing at 50 cm), and a Tobii EyeX eye-tracker, which was attached to the bottom of the screen by magnets. (B) The underlying test grid, in retinal coordinates (though note that actual locations could deviate from the underlying grid, as shown in Fig. 2). (C) An example trial sequence, in which a stimuli are presented relative to the current point of fixation, and the eye-tracker determines whether the participant moved their eyes toward the target. See body text for details.

The equipment for the Eyecatcher visual field test is shown in Figure 1A. Stimuli were displayed on a Microsoft Surface Pro 3 (Microsoft, Redmond, WA): a portable “tablet computer,” containing a screen measuring 25.4 × 16.9 cm (28.5° × 19.2° at the viewing distance of ∼50 cm). Eye movements were recorded monocularly at 50 Hz using a Tobii EyeX eye-tracker (Tobii Technology, Stockholm, Sweden): a low-cost (∼$100), near-infrared, remote eye-tracker, with a precision of < 0.6°.^40^ Participants were sat approximately 50 cm from the screen. However, no chin rest was used, and participants were free to move their head. A constant stimulus size on the retina was maintained by scaling the screen-size of the stimulus trial-by-trial, based on the participant's current viewing distance (as measured by the EyeX eye-tracker, which also contains integrated head-tracking). The test was programmed in C# and R,^41^ using custom code.

**Figure 2:**
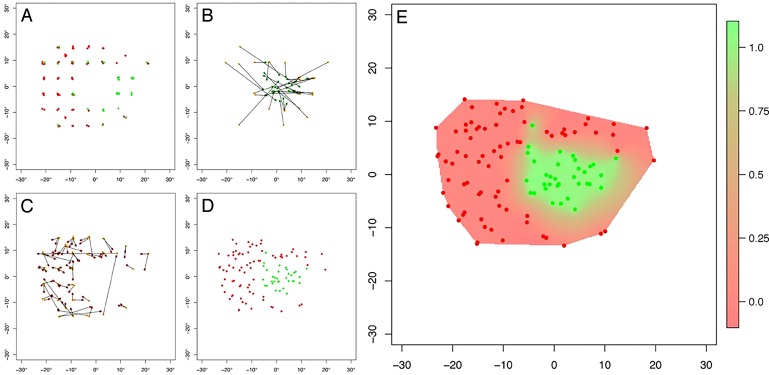
Computing performance using Eyecatcher. (A) Initially Eyecatcher attempted to present four stimuli at each of the 44 test locations in the underlying measurement grid (Fig. 1B). For each trial, the participant's response was scored as either a hit (green dots), or a Miss (red dots – NB: the location of each dot has been jittered slightly for visibility). (B, C) To correct for eye movements, after each trial (and irrespective of whether it was scored as a Hit or a Miss), the software searched back 200 ms, and used the participant's gaze location at this prior time point to replace the intended stimulus location (orange dots) with its true location (green/red dots). (D) The empirical test grid, after post hoc correction (same data as [A]). Due to the correction for eye movements, the precise shape and extent of this map is liable to differ on every test. (E) Finally, Kriging^42^ was used to interpolate the corrected data to provide a continuous heatmap indicating the Probability of Seeing at each location (red: highly unlikely; green: highly likely). Kriging was performed in R, using the Fields package.^43^

**Figure 3:**
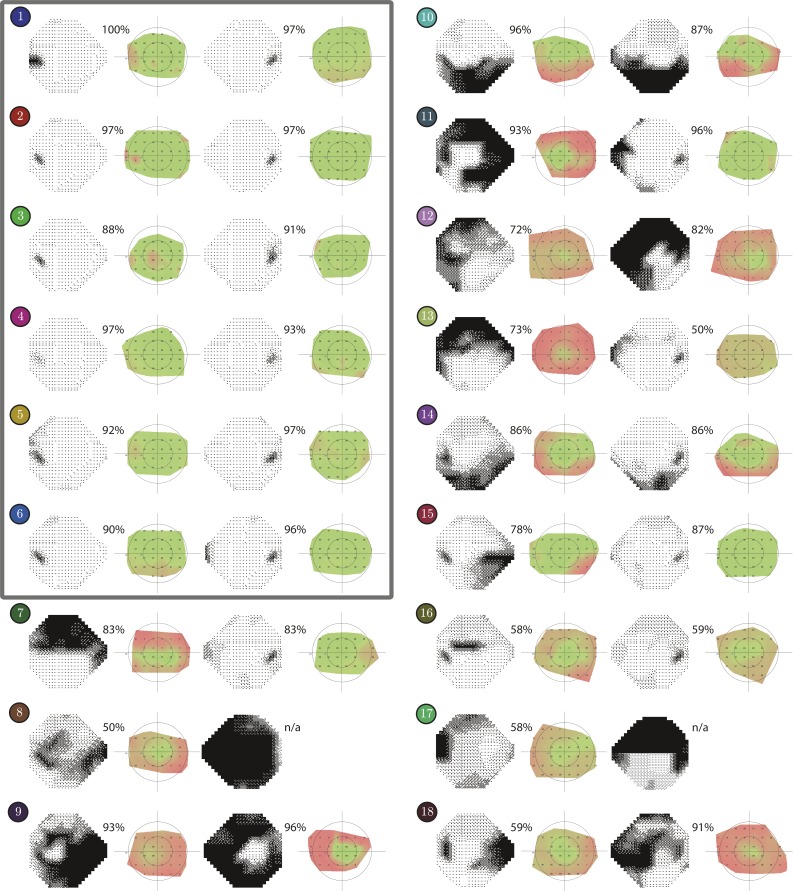
Concordance in pointwise sensitivity (Eyecatcher versus HFA). Results are shown for each individual eye, with the HFA Greyscale on the left, and the corresponding Eyecatcher Kriging surface on the right. If concordance was good, then black areas in the HFA Greyscale should appear as red areas on the Eyecatcher Kriging surface. Numbers indicate the percentage of concordant values at each 24-2 location present in both maps. Markers correspond to the list of individuals in the Table. Individuals 1–6 (gray rectangle) indicate healthy controls.

#### Task

Participants viewed the tablet monocularly (fellow eye patched), and were asked simply to “follow the dot.” Unlike SAP, they were not required to maintain fixation or press a button, since the eye-tracker was used to position stimuli and assess eye-movement responses (see below). Each eye was tested separately, in random order.

#### Stimuli

Stimuli were white Goldmann III (0.43°) circular spots, presented at a fixed intensity of 300 cd/m^2^ (equivalent to a ∼10 dB target in the HFA). Stimuli were presented against a white 31 cd/m^2^ background. The background was brighter than the HFA background (10 cd/m^2^) as the eye-tracker was observed to perform poorly under very low illumination. Luminance calibration was performed using a Minolta LS-110 spot photometer (Minolta Camera Co., Osaka, Japan).

#### Scoring Hits and Misses

A typical trial sequence is shown in Figure 1C. At the start of each trial, a stimulus was presented at a random location on an adapted 24-2 grid (see below). If, within 1500 ms of trial onset, the participant's gaze fell within 2.9° of the stimulus, then the trial was scored as a Hit (target seen). Otherwise, the trial was scored as a Miss (target unseen). If a trial was scored a Hit, then the current stimulus remained visible on the screen until the *next* point was fixated, but any previous points were hidden. From the user's perspective, they effectively “chased” a dot around the screen. If a trial was scored a Miss, then the current stimulus disappeared after 1500 ms; however, the last seen target remained visible (see Fig. 1C). The previous target was left visible as an optional “anchor,” to minimize unnecessary eye movements. However, this was not an obligatory fixation target, and participants were free to move their gaze if desired.

#### Measurement Grid

The underlying measurement grid is shown in Figure 1B. It consisted primarily of points from the standard 24-2 that could be fit within the view angle of the screen, along with four additional points at 〈±10°, ±10°〉. The four most central points from the 24-2 grid were omitted, as the spatial imprecision of the eye-tracker meant that measurements at these locations would be unreliable. The test attempted to test each location four times. Note, however, that because participants were free to move their eyes, and since stimuli were presented for relatively long duration (1500 ms), it was possible for participants to “cheat” by searching for the target. We developed a novel solution to account for this, as detailed in Figure 2. However, this resulted in the underlying test grid becoming irregular (see Fig. 2E).

#### Outcome Measures

The primary outcome measure was a map of hit rates, which can be interpreted as “probability of seeing” values. This map was constructed after the test was complete by using a technique known as Kriging to interpolate between responses across individual trials/locations^42^ (see Fig. 2). A summary measure of this map was also generated by mean-averaging the expected hit rate value at each of the discrete 24-2 locations present within the Kriging surface. The resultant metric, “Mean Hit Rate,” can be understood as the average amount of “greenness” in the plot, and is potentially comparable to HFA's MD metric.

#### Analysis: Comparison With HFA

We validated Eyecatcher against the HFA (the Reference Standard) in three ways. First, we compared visually the HFA grayscales against the Eyecatcher maps. Second, we computed pointwise concordance between the tests for defects at each of the HFA locations contained within the Eyecatcher surface. A location was defined as concordant if both “Sensitivity_HFA_ < 25 dB” and “Hit Rate_Eyecatcher_ < 0.5” (or the inverse), where 25 dB was an arbitrary threshold, but one that we believed most clinicians would regard as clinically significant. We then computed the percentage of concordant points for each eye. Note that the two tests do not measure the same thing: Eyecatcher measures hit rate (frequency of seeing) for a fixed threshold stimulus, whereas the HFA measures thresholds (just noticeable differences). There are therefore no directly equivalent cutoff points for the two tests. However, the present findings were qualitatively unchanged when values other than < 25 dB and < 0.5 were used. Third, we estimated the degree of correlation between the summary measures of visual field loss from Eyecatcher (Mean Hit Rate) and HFA (MD).

#### Analysis: Usability

Participants were given a short “usability” questionnaire, in which they were asked to assess both the HFA and Eyecatcher along five dimensions. Each question consisted of a statement (“I found the test… enjoyable, easy, tiring, hard to concentrate on,” and “I understood what was required”) to which participants were asked to rate their agreement based on a five-point Likert scale.

## Results

Test results for all individuals are shown in Figure 3. By inspection, it can be seen that there was good separation between patients (ID 7–18) and healthy peers (ID 1–6), and also between those eyes with mild and severe impairment (e.g., see ID 7, 11, and 13). These differences were confirmed statistically. Thus, there were significant differences in mean hit rate (“amount of greenness”) between the eyes of patients and controls (Between subjects *t*-test; *P* < 0.001) and also between patients' eyes with moderate (MD < 12 dB) and severe (MD > 12 dB) field loss (*P* < 0.001).

Within a single eye, Eyecatcher was also able to localize scotomas with reasonable spatial precision. Note, for example, the spared central vision in IDs 9 and 11, and the lower hemifield loss in IDs 10 and 14. In some cases, however, Eyecatcher did appear to overestimate the spread of visual field loss (e.g., IDs 13, 16, and 18).

**Figure 4:**
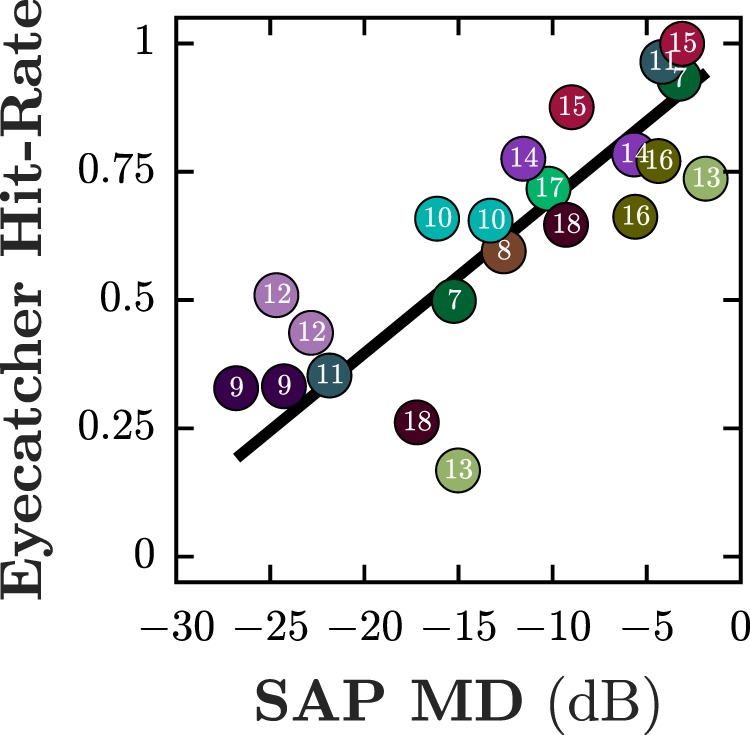
Agreement in overall sensitivity (Eyecatcher Mean Hit-Rate versus HFA MD value). Each data point represents a single eye from a patient (N = 22). The solid line shows the best fitting geometric-mean regression slope (i.e., “error-in-both-axes regression”).

Statistically, concordance between visual field defects on Eyecatcher field maps and HFA grayscales was generally good. Median (quartiles) percentage of concordant points was 83% (59%, 91%) in the 22 patient eyes, and 97% (92%, 97%) in the 12 eyes from visually healthy peers. As shown in Figure 4, there was also a good correlation between summary measures of visual field loss from Eyecatcher and HFA MD in patient eyes (Pearson Correlation: *P* < 0.001, *r^2^* = 0.64).

In terms of completion rates, all participants completed the HFA exam in both eyes (100% completion). However, two patients were only able to complete Eyecatcher in one eye (92% completion). In one case (ID 8/R), the vision loss was so severe that the patient was unable see to complete the initial eye-tracker calibration. In the other case (ID 17/R) the eye was highly myopic (−12.50/+2.00 × 90) and the eye-tracker was unable to track it reliably.

Eyecatcher was faster to complete than the HFA. In patients, mean (CI_95%_) test durations for Eyecatcher were 5.1 minutes (4.6, 5.5), versus 6.9 minutes (6.5, 7.5) for HFA (Pairwise *t*-test: *P* < 0.001). In healthy peers, test durations were 3.4 minutes (3.2, 3.5) for Eyecatcher, versus 4.8 minutes (4.6, 5.2) for HFA (Pairwise *t*-test: *P* < 0.001).

**Figure 5:**
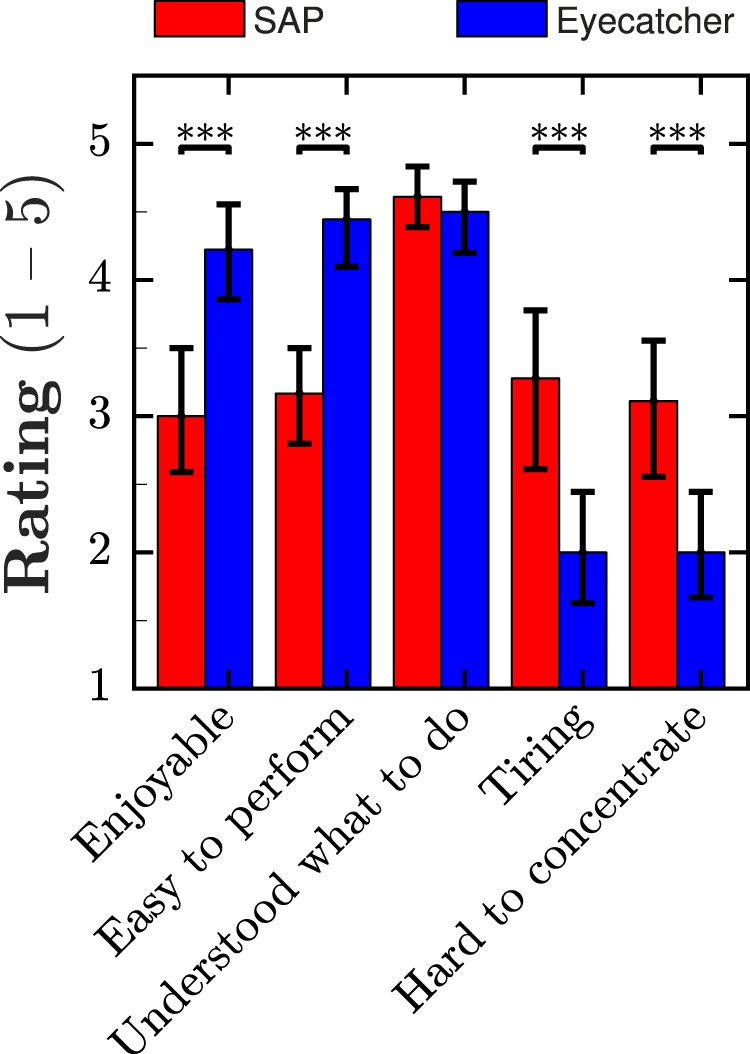
Group-mean [± 95% confidence interval] usability scores for the new test (Eyecatcher: blue) versus traditional perimetry (HFA: red): 1, strongly disagree; 5, strongly agree.

As shown in Figures 5, participants rated Eyecatcher more enjoyable, easier to perform, less tiring, and less hard to concentrate on than SAP (all *P* < 0.001). There was no difference in task-comprehension (*P* = 0.495), which was high for both tests—though it may be worth noting that participants already had previous experience of HFA testing.

## Discussion

Current SAP devices are not well-suited for glaucoma case-finding, due to being bulky, expensive, and often challenging to administer.^3^ The present study proposes and evaluates a novel alternative (“Eyecatcher”): a portable, suprathreshold eye-movement perimeter, which uses an inexpensive “clip-on” eye-tracker to detect eye movements toward successive targets (Goldmann III white lights). Unlike traditional SAP—or alternatives such as Frequency Doubling Technology,^44^–^46^ Flicker Perimetry,^47^ or Rarebit Perimetry^48^–^50^—this new approach requires minimal task-instructions, and does not require the participant to press a button or maintain fixation on a central marker. And unlike other eye-movement perimeters,^12^–^17^ Eyecatcher is intended primarily as a case-finding tool, and can be deployed using only a portable tablet-computer and low cost eye-tracking device (∼$100). This makes it potentially well-suited to rapidly identifying cases of suspected visual field loss, even in traditionally difficult to test populations.

Overall, the results showed that the approach is feasible, and that Eyecatcher shows promise as a rapid case-identification tool for visual field loss. Most individuals were able to complete the test with minimal difficulty, and the results showed good concordance with values derived from SAP (HFA). Our participants also reported a clear preference for Eyecatcher over SAP and this is particularly noteworthy. We consider each of these findings in turn, and discuss important caveats.

In terms of completion rates, all participants were able to perform the Eyecatcher test in at least one eye. However, two eyes with severe loss could not be tested: one because their vision was too poor to see the screen (ID 8/R), and the other because the eye-tracker was unable to track their eye through the patient's −12.5 diopter lenses (ID 17/R). Failures of this kind are not a substantial concern for our proposed case-finding device, since individuals with such severe vision loss would be expected to be receiving care already. However, the fact that eye-movement perimetry is not always possible would be important to bear in mind if considering it as a like-for-like replacement for current SAP devices.

There was good consistency between the results of Eyecatcher and HFA. Although the two tests are not equivalent (Eyecatcher measures hit rate for a fixed threshold stimulus, whereas the HFA measures detection thresholds), overall loss was nevertheless strongly correlated between the two tests (Fig. 4), and there was also good concordance between defects at individual pointwise locations (∼84%). Concordance was particularly high in healthy controls (95%), with relatively few locations misclassified as defects. In patients, concordance scores were still reasonable (78%), but the precise shape and location of the field loss was not always perfectly preserved, and there was in particular a tendency for the spatial extent of the vision loss to be overestimated. This could be due to a number of factors, including the limited spatiotemporal precision of the eye-tracker, or imperfect gaze-calibration. We believe, however, that the current implementation represents a good compromise between accuracy, cost, and test duration. For example, even without any refinement this feasibility study has illustrated that Eyecatcher can reliably detect moderate visual field loss, which is important for effective case finding.

In terms of usability, participants rated Eyecatcher as more enjoyable, easier to perform, and less tiring than SAP, and found it less hard to concentrate on than SAP. Participants also reported good task-comprehension on both tests (though this was to be expected in the case of SAP, as they all had existing prior experience of performing the test). These findings are particularly important and encouraging, because effective case finding depends on ease-of-use. The results are also consistent with a recent study by McTrusty and colleagues,^14^ which also found eye-movement perimetry to be more comfortable than traditional SAP, most likely due to the participant being able to move their eyes and head during the test.

In terms of test duration, Eyecatcher was faster than the HFA (5.1 vs. 6.9 minutes). However, the difference was modest considering the fact that Eyecatcher used a smaller grid, and used fixed-luminance stimuli (i.e., is a suprathreshold test, whereas the HFA estimates exact thresholds). Furthermore, test durations were substantially longer than other proposed screening measures, such as Frequency Doubling Technology (∼1 minute^46^,^47^,^51^,^52^). That test durations were not shorter in part reflects the fact that Eyecatcher required additional time to calibrate the eye-tracker, and also the fact that each target location was tested multiple times (four) to compensate for potential eye-tracking error. However, the relatively long test duration (∼5 minutes) is a limitation of the present test. We are currently exploring ways to reduce tests times, including improved calibration algorithms, and more efficient testing algorithms: for example, a single response may be sufficient if it is consistent with previous responses to neighboring locations. It is also worth noting that the intuitive nature of eye-movement perimetry saves time in a way that is not captured by traditional test duration metrics. Thus, with SAP, substantial additional time is required to explain the test and position the patient appropriately. With Eyecatcher, these requirements are largely eliminated, potentially allowing for more substantial time savings overall.

Further work is required to establish decision boundaries for the present test. Thus, it was encouraging that, even by casual inspection, there was a clear difference between healthy and affected eyes (Fig. 3), with minimal “false positive” red areas in the healthy controls. In the longer term though, for a test to be of practical utility there must exist a clear, formal procedure for mapping any results to the appropriate clinical decision: in this case, whether or not to refer the patient for further testing. The most straightforward way to do this would be to establish a “traffic light” system (mild / moderate / severe loss) based on normative limits. Alternatively, machine learning could be used to detect abnormal field-loss patterns, using the same basic techniques as those used elsewhere for classifying retinal images.^53^,^54^ Of course, in either case, a much larger sample of patients will be required.

A potential limitation of the present device is its restricted spatial range (the majority of stimuli were presented within ±15° horizontal, and ±9° vertical). In practice, the spatial range appeared sufficient to reliably discriminate between cases of glaucoma and age-similar controls, and this is consistent with a growing body of work indicating substantial central and paracentral impairments in glaucoma.^55^,^56^ It is conceivable, however, that individuals with a localized defect in the far periphery might be missed using the present device. Furthermore, it is important to note that the test was conducted under laboratory conditions. It remains an open question how well the test will perform in real clinical environments where, for example, one has less control over the ambient lighting conditions and the presence of potential distractors. Equally, it is not possible to assess at present how robust the test is to operator error, and whether, for example, rigorous training is required to ensure that the screen and eye-tracker are positioned correctly (e.g., in order to maintain an appropriate viewing angle and accurate determination of gaze). Finally, it is also important to note that the present study used a self-selecting subset of individuals who are likely to be relatively compliant and motivated. It remains to be seen how robust Eyecatcher is when applied to a larger, more diverse cohort, including, in particular, individuals who may be highly inattentive or malingering. It would also be instructive to evaluate the device with other conditions associated with visual field loss, such as diabetes, retinal dystrophies, and neurological disoders.^57^,^58^ We are currently investigating all of these factors by undertaking a more extensive evaluation of the device in an everyday clinical environment. We are also encouraged by the fact that many of these potential concerns can also be addressed in future by emerging technologies. For example, the same basic test could be deployed on a VR headset with an enclosed screen and integrated eye-tracking. This would allow extremely wide fields of view, complete control over ambient lighting, and real-time tracking of head-position, eye-position, gaze, and pupil size—all of which could be used as potential biomarkers for attentiveness/compliance.^59^,^60^

In terms of potential applications, Eyecatcher is primarily intended as a rapid, portable case-finding device, for use in community settings where specialist equipment/expertise is unavailable. In this respect, the fact that Eyecatcher is quick, cheap, does not require any physical contact with the participant, and does not require any explicit task instructions, makes it particularly attractive. Its portability and low cost also makes it well-suited to other situations where traditional SAP is unaffordable or impractical, for example for use in home monitoring,^20^ or in developing countries. Finally, this work spotlights the principle of using inexpensive eye tracking technology to “automatically” assess vision, leading to examinations that are far less demanding, and require less co-operation from the person being examined. Notably, the same basic equipment can also be used to test other aspects of vision. For example, by replacing spots of light with gratings of variable spatial-frequency, acuity can be measured.^10^ This approach could be particularly useful for populations who are currently hard to test, such as children, the very old or infirm, stroke patients, or individuals with cognitive impairments.
